# Integrated dry hole, seismic, and structural analysis improves hydrocarbon exploration in the Egyptian Northern Red Sea basin

**DOI:** 10.1038/s41598-026-61167-5

**Published:** 2026-07-15

**Authors:** Amr M. Eid, Mustafa Hassan, Mohammed Amer, Walid M. Mabrouk, Ahmed Metwally

**Affiliations:** 1 Geophysics Department, Faculty of Science, Cairo University, Giza, 12613 Egypt; 2 Ganoub El Wadi Petroleum Holding Company, Nasr City, 11765 Egypt

**Keywords:** Dry hole analysis, Petroleum system assessment, Northern Red Sea Region, Source analysis, Geochemical analysis, Energy science and technology, Solid Earth sciences

## Abstract

This study evaluates the hydrocarbon potential of Egypt’s Northern Red Sea through an integrated workflow combining geochemical, petrophysical, and seismic datasets. Dry Hole Analysis (DHA) was conducted on 14 wells, with detailed investigation of three representative failures (RSOX-94-1, QUSEIR_B_1X, and MIKAWA-1) to assess the relationship between structural trapping and petroleum system effectiveness. Geochemical results indicate variable source rock potential, with Total Organic Carbon (TOC) values ranging from 0.3% to 1.2%, corresponding to poor-to-fair source quality. These values are interpreted as residual organic richness due to the advanced thermal maturity of the Lower Miocene succession. Optical kerogen analysis reveals a predominance of woody kerogen (80–90%) with minor herbaceous components, indicating a mainly gas-prone petroleum system with limited localized oil potential. Thermal maturity data show a complex burial history, with Tmax values suggesting immature to early mature conditions at shallower depths (~ 2100 m), whereas vitrinite reflectance (Ro) values between 2.04% and 3.49% indicate overmature conditions within deeper Lower Miocene intervals. Petrophysical evaluation identifies reservoir intervals within the Belayim, Kareem, and Rudeis formations, characterized by average porosities of 6–9%, although reservoir continuity is constrained by facies variability and syn-rift structural compartmentalization. Seismic interpretation reveals widespread fault-controlled traps analogous to productive rift basins, but quantitative failure analysis indicates that trap integrity and hydrocarbon charge represent the principal exploration risks. RSOX-94-1 failed primarily due to fault-related seal breach and hydrocarbon leakage, whereas MIKAWA-1 was limited by insufficient hydrocarbon charge despite the presence of a valid structural trap. The integration of geochemical screening, reservoir characterization, and structural risk assessment provides a refined framework for frontier exploration in the Northern Red Sea and highlights key geological factors controlling exploration success in this underexplored rift basin.

## Introduction

The Red Sea has long been a focal point for scientists investigating the rifting of Earth’s lithosphere, offering a unique natural laboratory for understanding extensional tectonics and the evolution of hydrocarbon-rich passive continental margins. Once connected, the coastlines of Africa and Arabia can almost be realigned, except for a slight overlap at the Afar region, a key site of volcanic activity. The central and southern areas of the Red Sea feature a distinct submerged rift valley, while the northern section transitions into a series of deep basins containing brine pools and volcanic structures. Along the coastline, large tilted fault blocks dominate the landscape, with similar formations identified offshore through seismic reflection data. The region is highly seismically active, particularly at the intersection of the rift with the transform fault in the Gulf of Aqaba, further emphasizing its tectonic significance^1^.

Extensive research in the Red Sea and Gulf of Suez has significantly advanced the understanding of continental rifting, extensional faulting, orthogonal rift systems, and sedimentary basin evolution. These studies have provided important insights into sedimentation processes in continental and marine settings, the interaction between magmatism and crustal extension, and the mechanics of normal faulting^2^ Over the past five decades, a substantial amount of geophysical and geological data, including wellbore data reaching crystalline basement, aeromagnetic surveys, seismic refraction, and seismic reflection datasets, has been acquired across the region. Although these datasets have considerably improved understanding of the basin architecture, they often provide fragmented views of the crustal structure and tectonic evolution, with interpretations from localized studies commonly extrapolated to broader regional scales.

The Red Sea is now widely regarded as a global reference area for studying continental rift systems, particularly those associated with hydrocarbon-bearing passive margins^3^. The Egyptian Red Sea margin, extending approximately 650 km and representing nearly 30% of the total basin length, preserves some of the most complete structural and stratigraphic records associated with Red Sea rifting^4,5^. These preserved rift-related structures and sedimentary successions make the Egyptian margin a critical region for evaluating both geodynamic evolution and hydrocarbon prospectivity. However, despite the presence of favorable tectonic settings analogous to the prolific Gulf of Suez petroleum system, hydrocarbon exploration success in the Egyptian Northern Red Sea remains limited compared to neighboring sectors in Saudi Arabia and Yemen.

The Northern Red Sea is characterized by intense seismic activity and complex extensional tectonics that generated numerous tilted fault blocks, half-graben basins, and structurally controlled depocenters favorable for hydrocarbon entrapment^6^. Nevertheless, one of the principal exploration challenges in the region is the presence of extensive Miocene evaporitic sequences, which significantly degrade sub-salt seismic imaging and complicate the delineation of reservoirs, seals, and structural traps. Consequently, uncertainty in trap integrity, reservoir distribution, and hydrocarbon charge remains a major exploration risk throughout the basin.

To address these challenges, this study integrates seismic interpretation, petrophysical evaluation, and geochemical investigations to assess the geological controls governing hydrocarbon accumulation in the Northern Red Sea. Particular emphasis is placed on evaluating source rock quality and maturity, reservoir characteristics, structural trapping mechanisms, and seal efficiency to refine subsurface interpretations and improve petroleum system understanding^7,8^. By combining these multidisciplinary datasets, the study aims to reduce exploration uncertainty and support more effective future drilling strategies in this frontier basin.

A key component of this research is the Dry Hole Analysis (DHA) conducted on fourteen exploration wells drilled in the Northern Red Sea, the majority of which failed to encounter commercial hydrocarbon accumulations. The selection of these wells was based on data availability, geographic distribution across the basin, and their representation of different structural and stratigraphic settings encountered during exploration campaigns. From this regional dataset, three wells (RSOX-94-1, QUSEIR_B_1X, and MIKAWA-1) were selected for detailed case-study analysis because they collectively represent the principal petroleum system failure mechanisms observed throughout the basin.

Specifically, RSOX-94-1 was selected as a representative example of seal and trap integrity failure, where fault-related breach of the top seal occurred despite the presence of moderate-quality reservoir intervals. MIKAWA-1 represents hydrocarbon charge and source maturity risk, where structurally valid closures were identified but insufficient source rock maturity limited hydrocarbon generation and migration. In contrast, QUSEIR_B_1X exemplifies reservoir-related failure, where favorable structural closure was present but effective reservoir facies were absent. The selection of these three wells therefore provides a systematic framework for investigating the dominant geological risks affecting exploration success in the Northern Red Sea rather than relying on generalized basin-wide interpretations.

Although Dry Hole Analysis (DHA) and petroleum system evaluation are widely applied in mature basins, their application in the Northern Egyptian Red Sea remains limited due to sparse well control, complex salt tectonics, and poor sub-salt seismic imaging. The novelty of this study lies in integrating seismic interpretation, petrophysical evaluation, Rock–Eval geochemistry, biomarker analysis, and well-based DHA within a unified framework to identify the dominant causes of exploration failure in three key wells from the Northern Red Sea. Unlike previous regional studies that focused primarily on structural interpretation or basin evolution, this work quantitatively links individual petroleum system elements (charge, reservoir, seal, and trap integrity) to specific dry hole outcomes. The study also highlights the impact of drilling additive contamination on geochemical interpretation and proposes an integrated quality-control workflow for evaluating uncertain datasets in frontier rift basins. These findings provide a practical exploration risk framework for future Red Sea drilling campaigns.

## Geological setting

**Fig. 1:**
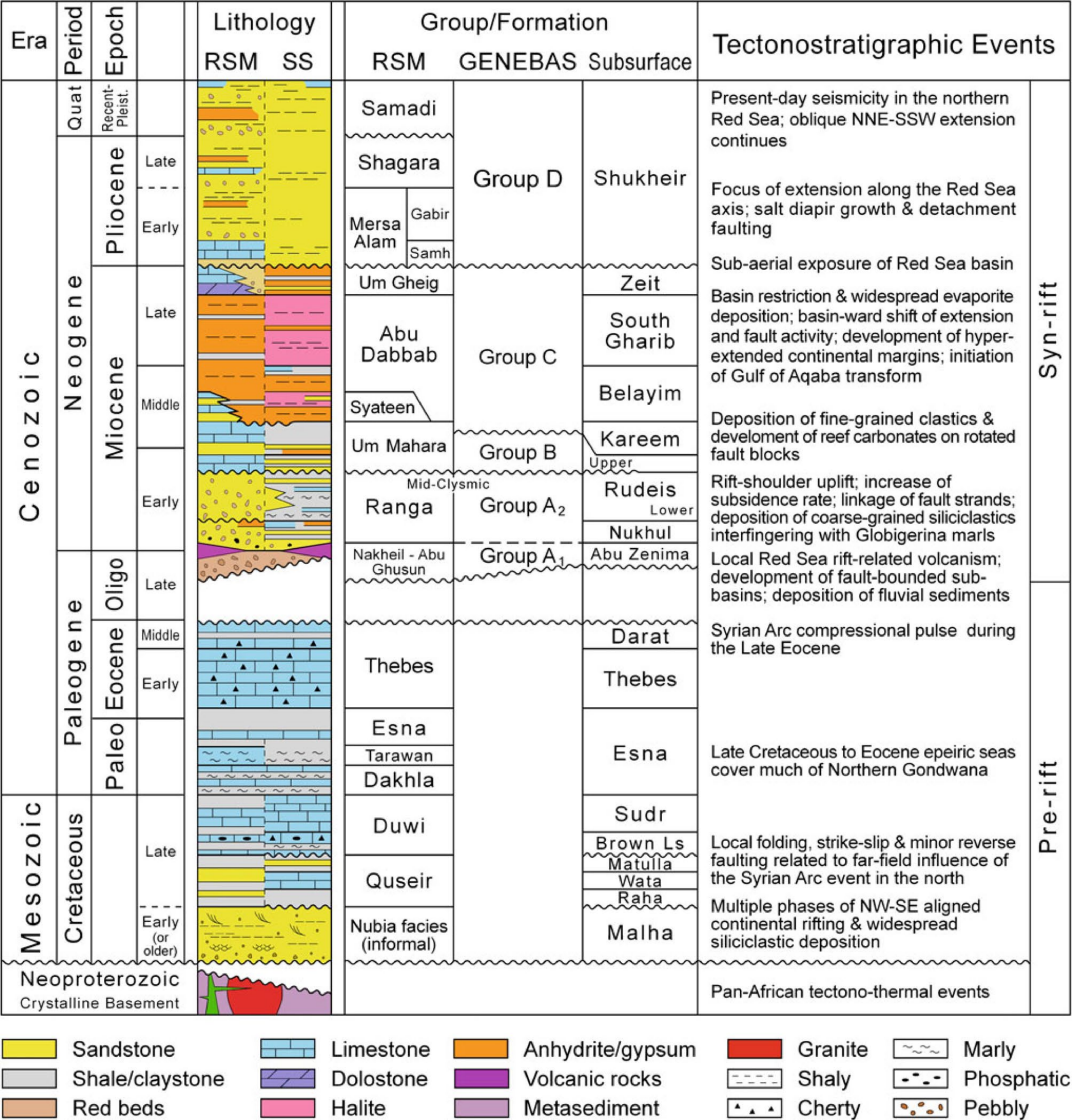
Comparison of the stratigraphic terminology commonly used along the Egyptian Red Sea margin (RSM) and in the subsurface (SS) of the Red Sea and Gulf of Suez^9^.

The geological evolution of the Northern Red Sea is marked by a transition from a passive margin setting during the Cambrian to the Eocene into a structurally complex rifted basin. Early sedimentation was dominated by siliciclastic deposition along the Neo-Tethyan margin, forming extensive sandstone reservoirs within the Nubian Group. A significant transgressive event in the Cenomanian resulted in the accumulation of mixed carbonate-siliciclastic sequences, including key source rocks such as the Duwi and Dakhla formations (Fig. 1). During the Oligocene, a global sea-level fall led to widespread continental conditions and the deposition of the Abu Zenima (Tayiba) Formation, coinciding with the initiation of volcanic activity in the Afar region^1,2,9^.

This volcanism extended northward, influencing the structural framework of the Arabian and Egyptian margins. The early phases of rifting in the Red Sea, initiated during the Early Miocene, were accompanied by the formation of half-graben basins and the deposition of marine sequences, including organic-rich shales, marls, and sandstones of the Rudeis Formation. This period also witnessed the development of rift-parallel fault trends intersected by complex cross-fault geometries, particularly along the offshore Egyptian Red Sea margin, which exhibits structural similarities to the southern Gulf of Suez^6,10,11^.

**Fig. 2:**
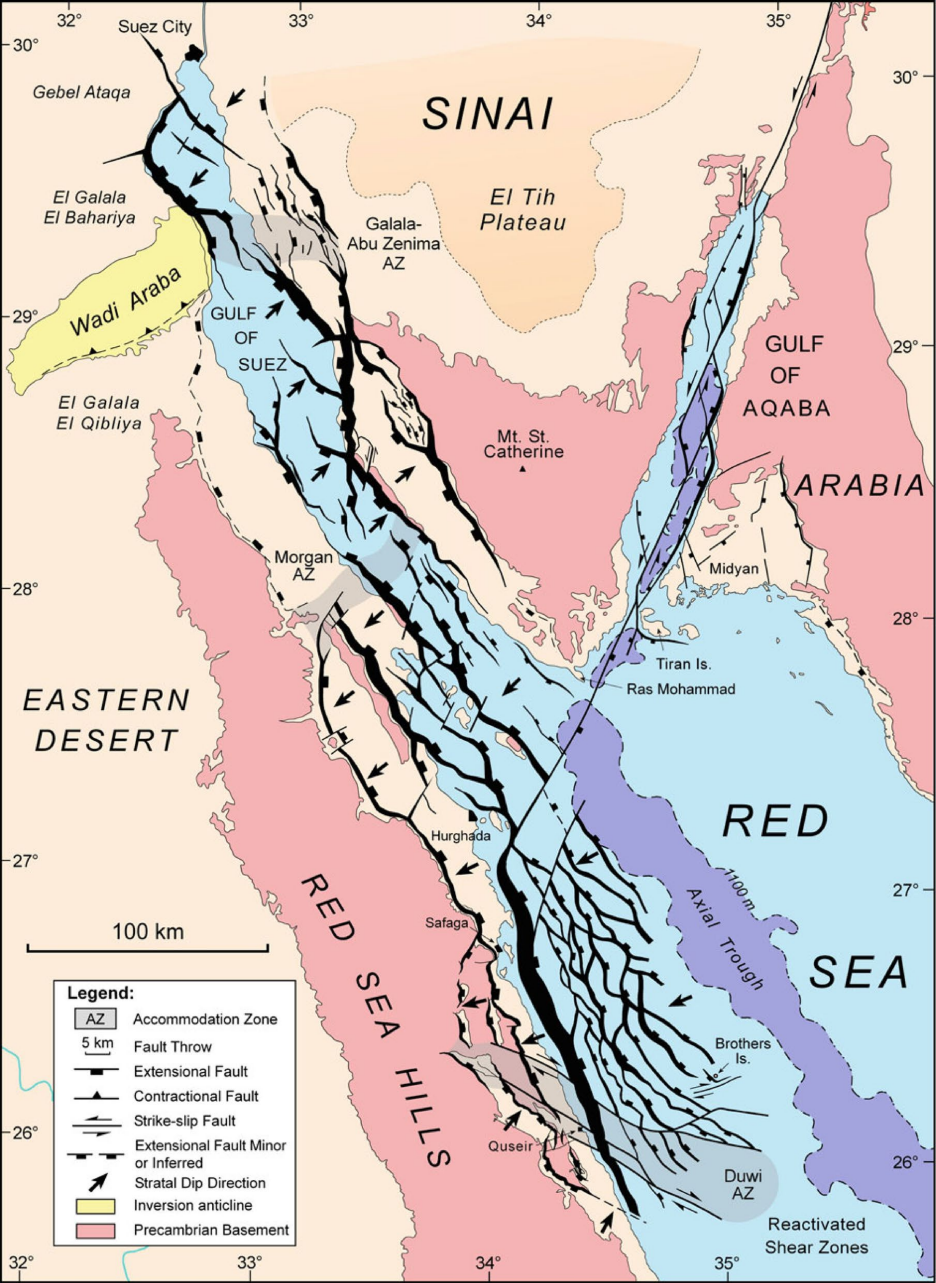
Compilation of fault patterns for the northern part of the Egyptian Red Sea margin and the Egyptian offshore Red Sea^2^.

By the Middle Miocene, rift-related uplift and basin subsidence resulted in the deposition of siliciclastic and evaporitic sequences, with the Kareem Formation representing a prominent evaporite unit. The Late Miocene was characterized by significant tectonic reorganization, particularly with the activation of the Dead Sea-Aqaba Transform Fault. This induced left-lateral shear deformation, influencing the propagation of faults in the Northern Red Sea and contributing to the cessation of active rifting in the Gulf of Suez. The offshore Egyptian margin is structurally defined by two major extensional fault systems forming a series of NW to N-S trending half-graben basins (Fig. 2)^12–14^.

These structural features have been revealed through extensive geophysical surveys, including early 2D reflection seismic studies and later 3D seismic acquisitions conducted between 1999 and 2008. However, imaging deeper fault blocks remains challenging due to salt tectonics, with extensive salt domes, walls, and canopies obscuring underlying structures. The mobilization of these evaporites, initiated in the Middle to Late Miocene, has significantly influenced sediment distribution and the development of potential hydrocarbon traps^1,3^.

**Fig. 3:**
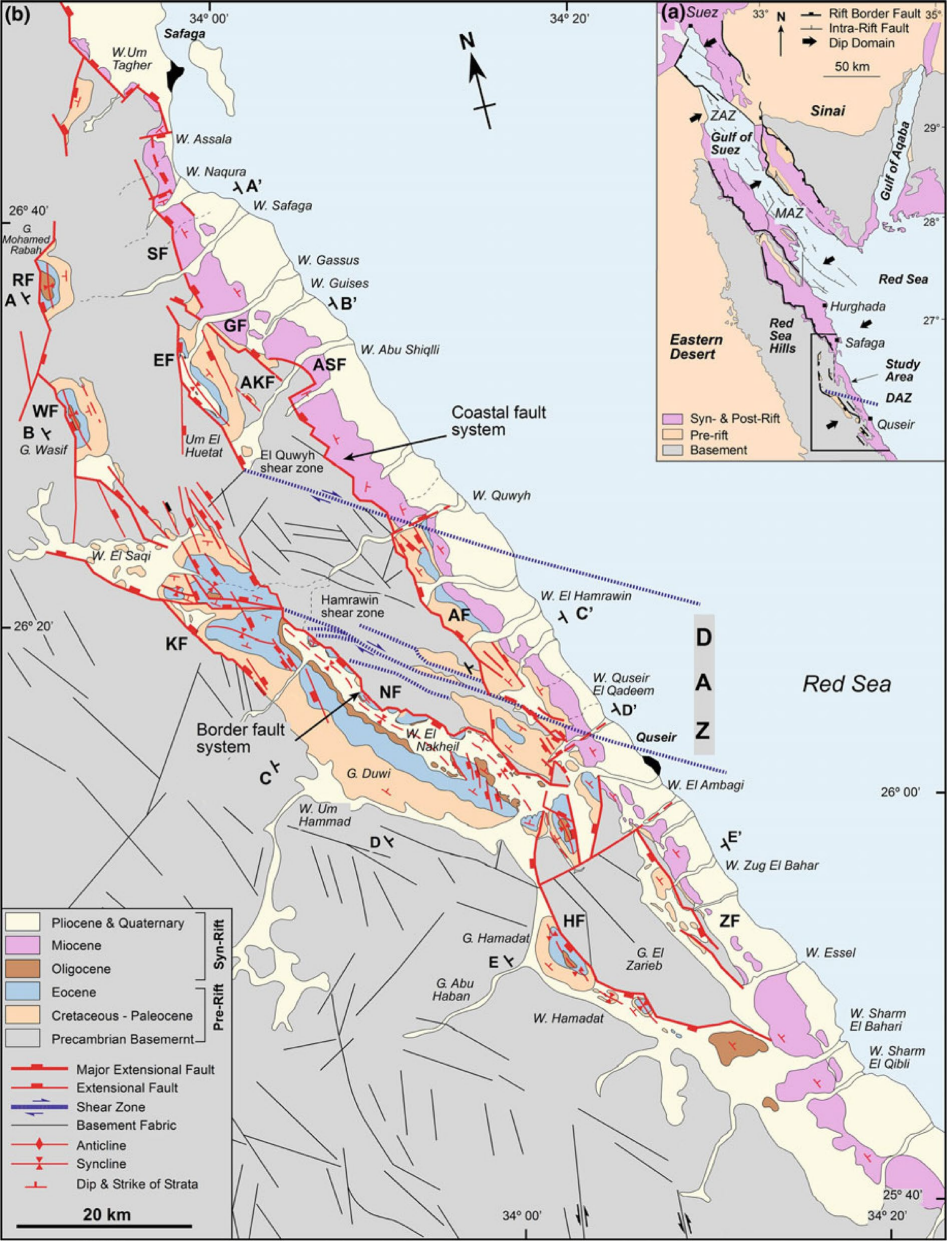
Regional distribution of basement, pre-rift, and syn-rift to post-rift strata and the location of the study area^2^.

The Pliocene marked the onset of seafloor spreading and oceanic crust formation, restoring fully marine conditions as the Red Sea connected with the Indian Ocean. The nature of the crust in the Northern Red Sea remains a subject of debate, with various models suggesting either episodic seafloor spreading or progressive mantle-driven extension. Pliocene-Recent sedimentation consisted of siliciclastic and carbonate deposits overlying Late Miocene evaporites, which continue to be structurally reworked by salt tectonics. The formation of salt diapirs, rim-synclinal basins, and associated structural traps plays a crucial role in hydrocarbon entrapment, yet exploration remains hindered by high seismic velocities and complex subsurface geometries (Fig. 3)^10,15,16^.

In addition, structural complexities associated with the offshore Egyptian margin, including a NE-dipping fault trend parallel to the Coastal Fault System, further complicate seismic imaging, particularly in environmentally sensitive nearshore areas. While no pre-rift strata have been encountered in existing offshore wells, exposures on Zabargad Island suggest potential pre-rift analogs. Continued advancements in seismic imaging and structural modeling are essential for refining hydrocarbon exploration strategies, mitigating the risk of dry wells, and enhancing the understanding of the Red Sea’s tectono-stratigraphic evolution^5,9,17^.

The Northern Egyptian Red Sea hosts a complex petroleum system influenced by source rock maturity, reservoir effectiveness, and structural dynamics that govern hydrocarbon formation, movement, and accumulation. This region holds significant exploration potential, supported by diverse geological formations with favorable characteristics for oil and gas generation^18,19^.

Key source rocks in the area span the Miocene and pre-Miocene, showing variations in thickness and maturity. Organic geochemical analyses, including Hydrogen Index (HI) and Tmax values, suggest that kerogen types range from oil-prone Type II to mixed Type II/III, indicative of both oil and gas potential.

Notable source formations include:

- Belayim Formation: Contains organic matter with Total Organic Carbon (TOC) up to 4% and high hydrocarbon generation potential.
- Kareem and Rudeis Formations: Exhibit comparable TOC values and pyrolysis characteristics, with oil-prone shales and marls.
- Older Units (Matulla, Sudr, and Duwi Formations): The Matulla Formation, in particular, presents exceptional richness, with TOC exceeding 3% in certain intervals, reaching up to 12% in localized sections.

Maturity studies indicate that Miocene source rocks in the Egyptian Red Sea are less thermally advanced than those in the Gulf of Suez, with deeper burial required to reach the oil window^1,3^. Thermal history reconstructions and vitrinite reflectance (Ro) values further delineate zones of oil and gas generation.

Reservoir potential is found within pre-rift Nubian sandstones, syn-rift clastic deposits, and carbonate systems, each exhibiting distinct properties favorable for hydrocarbon storage and flow^20^.

- Nubian Sandstones: These pre-rift reservoirs are composed of high-quality fluvial and deltaic sandstones, known for excellent porosity and permeability. Though their presence offshore Egypt is not fully confirmed, they are well-developed in Saudi Arabian counterparts.
- Miocene Syn-Rift Sandstones: These formations exhibit variable reservoir properties, with better quality found in well-sorted, coarse-grained deposits. Fault blocks may enhance permeability through diagenetic effects.
- Carbonate Reservoirs: Miocene carbonate platforms and reefal buildups, similar to those observed in the Midyan Basin (Saudi Arabia), display secondary porosity from karstification, improving their reservoir potential.

The structural framework of the region is dominated by salt tectonics, which has played a key role in trap formation. Hydrocarbon entrapment occurs in both structural and stratigraphic traps influenced by salt mobilization.

- Primary Seals: Miocene evaporites act as extensive regional seals, preventing vertical hydrocarbon migration. Thick anhydrite and salt layers are particularly effective in containing hydrocarbons within subsalt and intra-salt reservoirs.
- Secondary Seals: Fine-grained mudstones within Miocene sequences provide additional containment, enhancing trap efficiency.

Migration pathways are largely controlled by faulting and salt-related structures, facilitating hydrocarbon movement into suitable traps. Insights from the Gulf of Suez suggest similar trapping mechanisms in the Egyptian Red Sea, reinforcing its hydrocarbon potential.

## Materials and methods

**Fig. 4:**
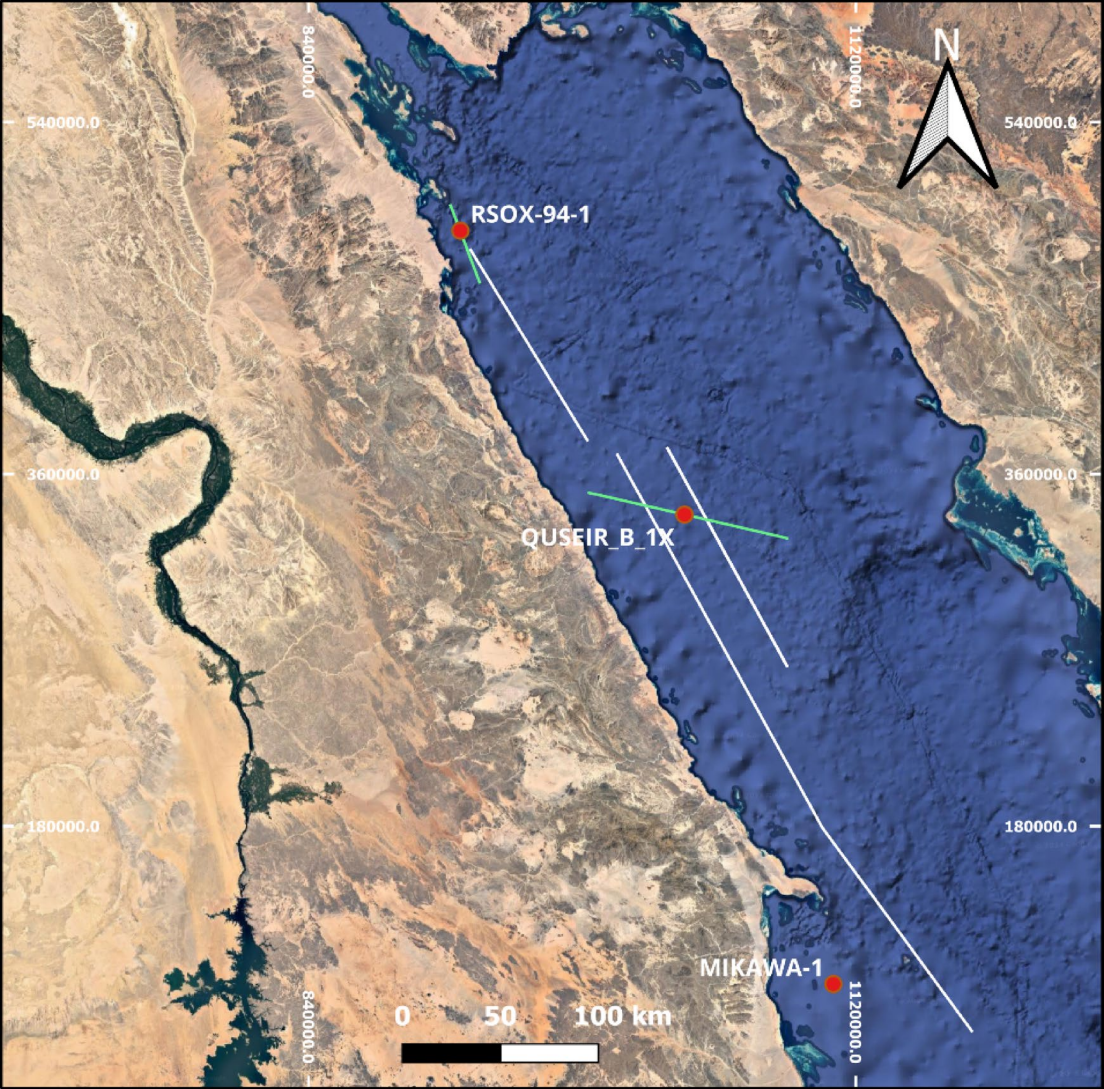
Location map of the study area showing the available seismic lines and wells. The satellite imagery was generated using QGIS 3.34.1 (https://qgis.org/).

**Fig. 5:**
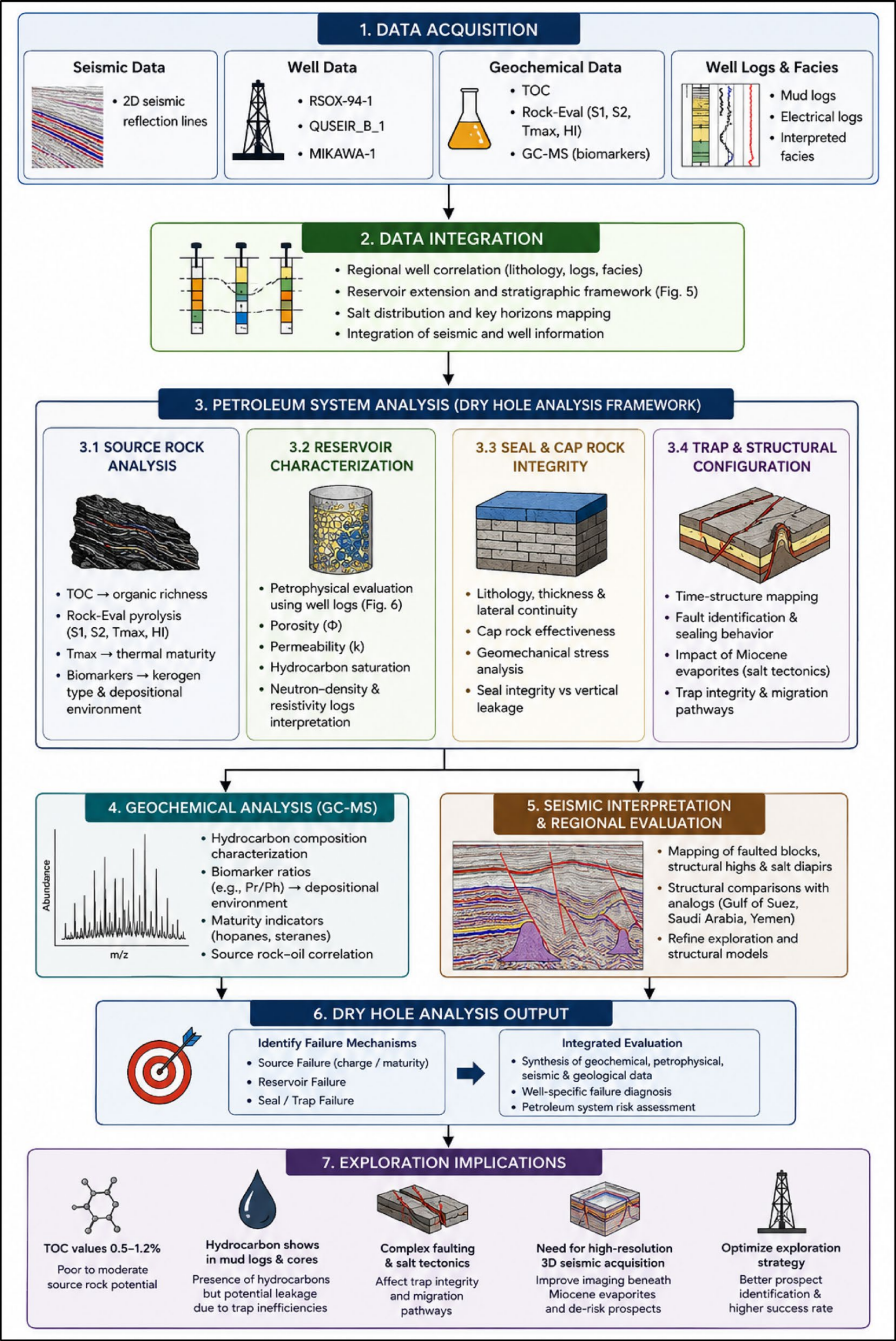
Workflow illustrates the integrated Dry Hole Analysis (DHA) approach combining seismic interpretation, geochemical evaluation, and petrophysical analysis to identify petroleum system failure mechanisms in the Northern Red Sea basin.

This study integrates seismic interpretation, petrophysical evaluation, geochemical analysis, and Dry Hole Analysis (DHA) to investigate the causes of exploration failure in three exploration wells from the Northern Egyptian Red Sea: RSOX-94-1, QUSEIR_B_1X, and MIKAWA-1 (Fig. 4). The adopted methodology follows an integrated petroleum system workflow combining seismic, geological, petrophysical, and geochemical datasets to identify the principal geological risks controlling hydrocarbon prospectivity. The detailed workflow adopted in this study is illustrated in Fig. 5.

The workflow consists of seven integrated stages: (1) data acquisition, (2) data integration and regional correlation, (3) petroleum system analysis using a Dry Hole Analysis (DHA) framework, (4) geochemical analysis, (5) seismic interpretation and regional evaluation, (6) integrated dry hole analysis output, and (7) exploration implications and prospectivity assessment.

### Data acquisition

Multiple datasets were integrated in this study, including 2D seismic reflection profiles, well data, geochemical analyses, and wireline log datasets. Three exploration wells (RSOX-94-1, QUSEIR_B_1X, and MIKAWA-1) were selected based on data availability and their strategic distribution within the Northern Red Sea rift system.

The geochemical dataset included Total Organic Carbon (TOC), Rock-Eval pyrolysis parameters (S1, S2, Tmax, and Hydrogen Index), and Gas Chromatography–Mass Spectrometry (GC-MS) biomarker analyses. Wireline datasets included gamma ray, density, neutron, resistivity, and mud logs. Lithological descriptions and interpreted facies distributions were also incorporated to constrain depositional environments and reservoir distribution.

### Data integration and regional correlation

**Fig. 6:**
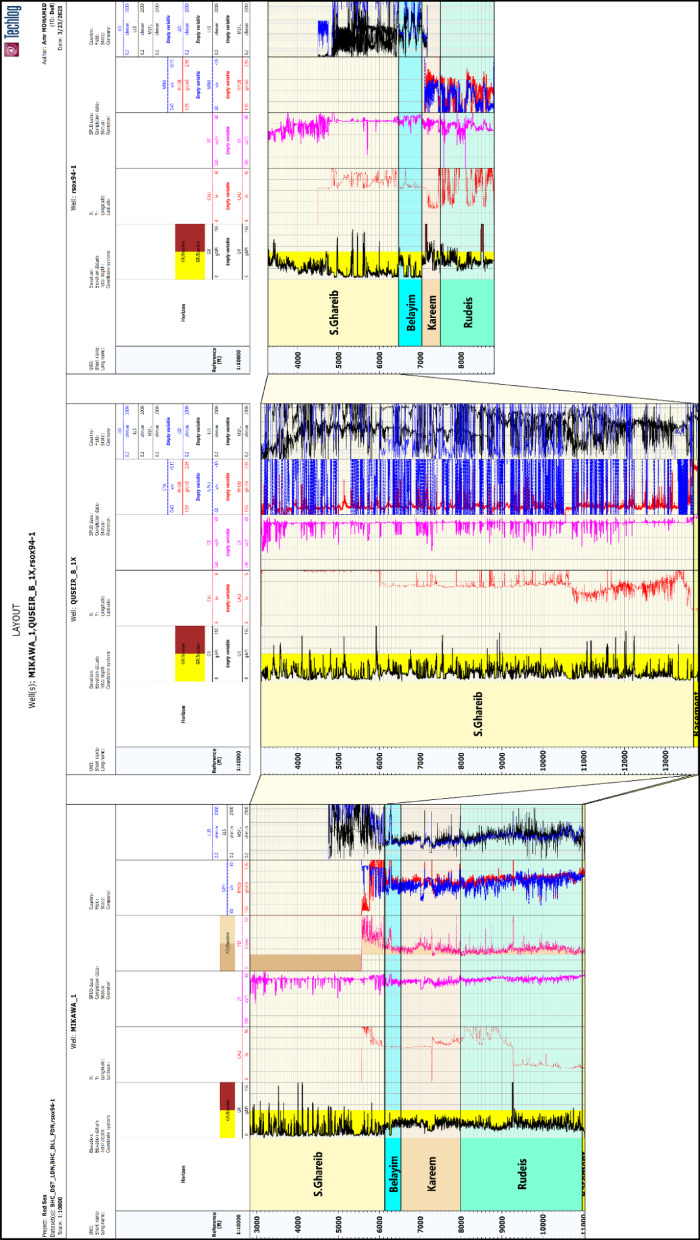
Well correlation panel illustrating the lateral continuity and thickness variation of the Miocene reservoir units across the study area, highlighting their spatial extent beneath the regionally extensive overlying evaporite sequences that provide an effective sealing interval.

Regional well correlation was performed using lithological descriptions, wireline log responses, interpreted facies, and seismic stratigraphic relationships to establish the regional stratigraphic framework of the study area (Fig. 6). Correlation focused on evaluating reservoir continuity, Miocene evaporite distribution, and key stratigraphic horizons across the Northern Red Sea basin.

Integration of seismic and well datasets enabled mapping of reservoir extension, salt distribution, and structural relationships between fault blocks and hydrocarbon-bearing intervals. This integrated framework was subsequently used for petroleum system evaluation and Dry Hole Analysis (DHA).

### Petroleum system analysis (Dry Hole Analysis framework)

A structured Dry Hole Analysis (DHA) workflow was applied using a petroleum system approach to identify the dominant causes of exploration failure in each well. The DHA framework evaluated the four principal petroleum system elements: source rock potential, reservoir quality, seal integrity, and trap effectiveness.

Each petroleum system element was qualitatively classified as effective, partially effective, or ineffective based on integrated geological, geophysical, petrophysical, and geochemical criteria. The dominant ineffective element was interpreted as the primary cause of dry hole occurrence.

#### Source rock analysis

Rock-Eval pyrolysis was conducted to evaluate source rock richness, kerogen type, and thermal maturity using TOC, S1, S2, Tmax, and Hydrogen Index (HI) parameters^21,22^. Biomarker and optical analyses were additionally used to characterize kerogen composition, depositional environment, and hydrocarbon maturity.

Because the analyzed samples were primarily derived from drill cuttings, a geochemical quality-control workflow was implemented to evaluate the influence of drilling additives on Rock-Eval and biomarker interpretations. A total of 100 samples from the interval 1900–4201 m were analyzed using TOC and Rock-Eval pyrolysis, while selected samples were further evaluated using optical kerogen analysis, vitrinite reflectance (Ro), solvent extraction, GC-MS, and isotopic analyses.

Contaminated samples were identified using multiple criteria, including anomalous or broadened S2 peaks, suppressed Tmax values below 400 °C, absence of kerogen cracking peaks, elevated S1 and Production Index (PI) values inconsistent with maturity trends, solvent extraction anomalies, visual detection of drilling additives during optical analysis, and biomarker distributions inconsistent with indigenous organic matter.

The contamination effect was particularly evident in several intervals, including samples at 2550 m, 2902 m, and 3001 m, where distorted S2 peaks and abnormal Tmax values indicated the presence of drilling additives rather than true kerogen cracking. These samples were considered fully compromised for Rock-Eval source-rock assessment and were excluded from quantitative interpretation. In contrast, samples that did not exhibit multiple contamination indicators and showed internally consistent Rock-Eval, optical, and biomarker characteristics were considered to retain interpretable geochemical signals and were therefore used in the final source-rock evaluation.

After filtering contaminated samples, the remaining dataset consistently showed poor source rock characteristics, with most S2 values below 0.5 kg HC/ton rock and generally low TOC values within the analyzed intervals. Sensitivity analysis further demonstrated that exclusion of contaminated samples did not significantly alter the overall interpretation of limited hydrocarbon generation potential and predominantly gas-prone Type III woody kerogen.

Although several samples displayed low Tmax values (< 400 °C), vitrinite reflectance measurements ranging from 2.04% to 3.49% confirmed an overmature thermal regime within the Lower Miocene section. The apparent inconsistency between Tmax and maturity indicators is therefore interpreted as an analytical effect related to drilling additive contamination rather than contradictory thermal evolution.

#### Reservoir characterization

1 $$\:{V}_{sh}=\frac{G{R}_{log}-G{R}_{min}}{G{R}_{max}-G{R}_{min}}\:$$

**Fig. 7:**
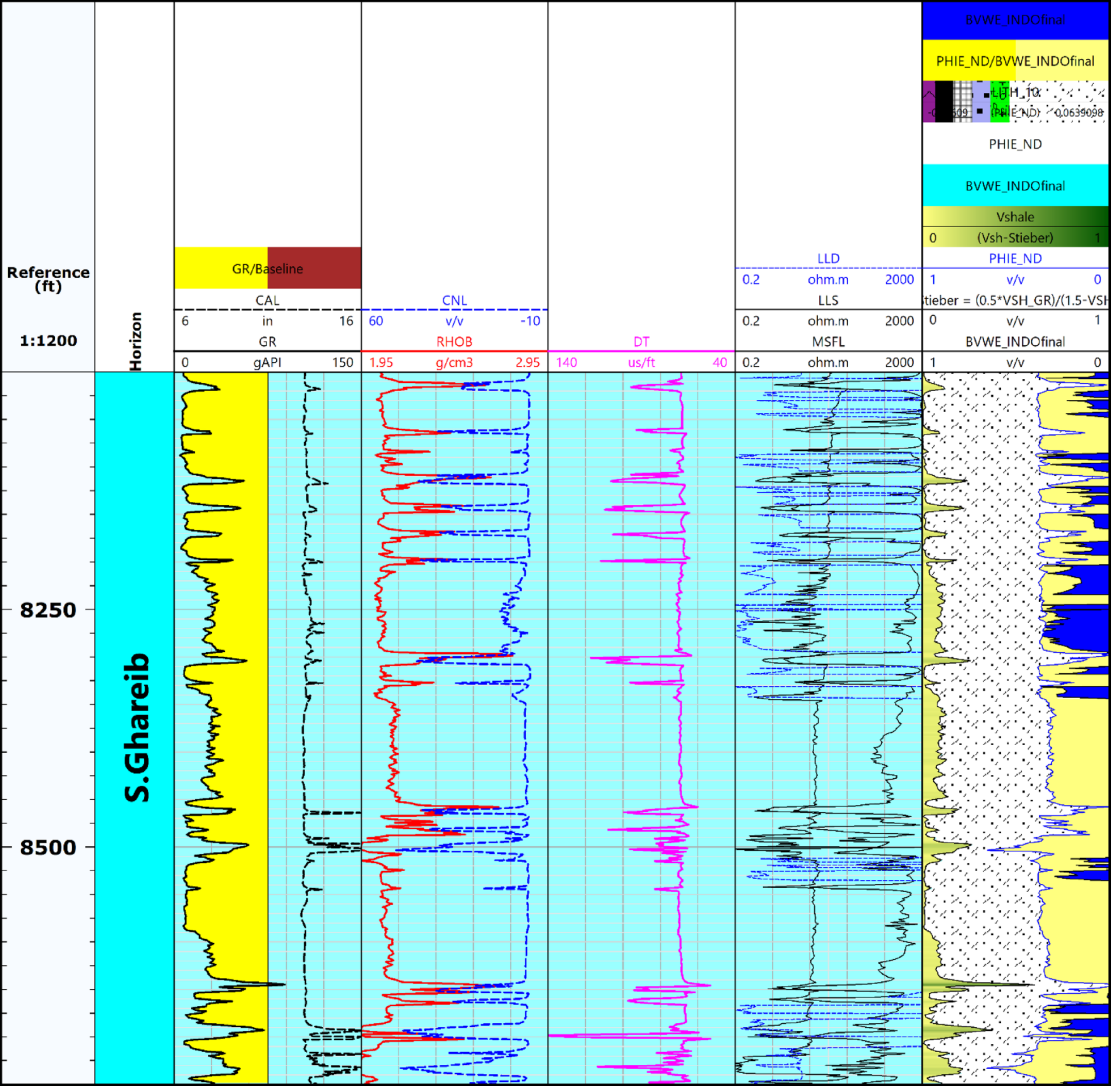
QUSEIR_B_1X formation evaluation.

Petrophysical evaluation (Figure. 7) was performed using gamma ray, density, neutron, and resistivity logs to estimate shale volume, porosity, and water saturation.

*Volume of Shale (Vsh)*

Shale volume was calculated from Gamma Ray (GR) logs using^23^:

2 $$\:{\phi\:}_{D}=\frac{{\rho\:}_{ma}-{\rho\:}_{b}}{{\rho\:}_{ma}-{\rho\:}_{fl}}\:\:$$

where $\:G{R}_{log}$is the measured gamma ray value, while $\:G{R}_{min}$and $\:G{R}_{max}$represent clean and shale responses, respectively.

*Porosity Calculation*

Density porosity was estimated using^24^:

3 $$\:{\phi\:}_{T}=\frac{{\phi\:}_{N}+{\phi\:}_{D}}{2}$$

Total porosity was calculated from neutron and density logs:

4 $$\:{\phi\:}_{eff}={\phi\:}_{T}-({V}_{sh}\times\:{\phi\:}_{sh})\:\:$$

Effective porosity was corrected for shale content using:

 Water Saturation (Sw) .

 Water saturation was estimated using the modified Archie equation^25^:

5 $$\:\frac{1}{\sqrt{{R}_{t}}}=\left[\sqrt{\frac{{\phi\:}^{m}}{a{R}_{w}}}+\frac{{V}_{cl}^{\left(\frac{1-{V}_{cl}}{2}\right)}}{\sqrt{{R}_{cl}}}\right]{S}_{w}^{n}\:$$

Net reservoir intervals were identified using cutoff criteria of effective porosity greater than 8%, water saturation lower than 60%, and shale volume less than 35%. These cutoff values were selected based on Miocene reservoir analogues from the Gulf of Suez and adjusted to account for burial depth and compaction effects within the Northern Red Sea. Hydrocarbon-bearing intervals were differentiated from water-saturated zones through integrated interpretation of neutron–density crossover responses and resistivity log characteristics.

#### Seal and cap rock integrity

Seal integrity was evaluated based on lithology, evaporite thickness, lateral continuity, and structural disruption by fault systems^26,27^. Emphasis was placed on the Zeit and South Gharib evaporite sequences, which represent the principal regional sealing units.

The effectiveness of cap rocks was further assessed through evaluation of fault-related leakage pathways and geomechanical considerations affecting vertical hydrocarbon migration. Relationships between hydrocarbon shows and fault geometries were incorporated into the DHA interpretation to evaluate seal effectiveness.

#### Trap and structural configuration

Structural interpretation focused on identifying tilted fault blocks, horst and graben systems, relay ramps, and fault-controlled trap geometries within the Northern Red Sea rift system. Time-structure mapping was applied to seismic datasets to delineate structural closures and evaluate trap integrity.

Particular emphasis was placed on assessing the impact of Miocene evaporites and salt tectonics on fault seal behavior and hydrocarbon migration pathways^1,28,29^. Structural configurations were integrated with petrophysical and geochemical observations to evaluate the effectiveness of potential hydrocarbon traps.

### Geochemical analysis (GC-MS)

Advanced Gas Chromatography–Mass Spectrometry (GC-MS) analysis was performed to characterize hydrocarbon composition and evaluate potential source rock affinity^30^. Biomarker ratios including pristane/phytane (Pr/Ph) were used to infer depositional conditions, while hopane and sterane distributions were evaluated to assess thermal maturity and hydrocarbon origin^22,30,31^.

Geochemical interpretations focused on distinguishing indigenous hydrocarbons from migrated hydrocarbons and identifying possible source rock correlations within the Northern Red Sea petroleum system.

### Seismic interpretation and regional evaluation

Seismic interpretation was conducted using 2D post-stack migrated seismic profiles calibrated with available well data. Key reflectors corresponding to the Zeit, South Gharib, Belayim, Kareem, and Rudeis formations were identified through well-to-seismic correlation using regional stratigraphic markers and synthetic well ties. Fault interpretation focused on mapping faulted blocks, structural highs, relay structures, and salt-related deformation features. Structural comparisons with productive Red Sea analogues in Saudi Arabia and Yemen, as well as Gulf of Suez reservoirs, were incorporated to refine exploration models and evaluate regional petroleum system similarities^32,33^.

Seismic quality decreases significantly beneath evaporite sequences due to signal attenuation and velocity distortion associated with salt tectonics. Consequently, interpretation of sub-salt structures relied heavily on well calibration and regional tectonic analogues.

### Integrated dry hole analysis output

The final DHA output integrated geochemical, petrophysical, seismic, and geological datasets to identify the dominant failure mechanisms controlling each dry hole. Failure mechanisms were classified into:

- Source rock failure (insufficient charge or maturity),
- Reservoir failure,
- and seal/trap failure.

This integrated interpretation enabled well-specific diagnosis of exploration failure and provided a petroleum system-based risk assessment framework for future exploration activities in the Northern Red Sea.

### Exploration implications

The integrated workflow indicates that the Northern Red Sea petroleum system is affected by poor to moderate source rock quality (TOC values ranging from 0.5 to 1.2%), localized hydrocarbon shows, complex faulting, and extensive salt tectonics. Although hydrocarbon shows confirm the presence of migrated hydrocarbons within the system, trap integrity and seal effectiveness remain significant exploration risks.

The study further demonstrates that improved imaging beneath Miocene evaporites through high-resolution 3D seismic acquisition is essential for reducing structural uncertainty and improving prospect identification. The integrated DHA framework developed in this study provides a practical exploration risk assessment approach for future drilling campaigns within the Northern Red Sea rift system.

## Results and discussion

### lithological descriptions of Key Formations in the Northern Egyptian Red Sea for the drilled wells

The lithological descriptions of key formations were derived from integrated interpretation of wireline logs (gamma ray, resistivity, density–neutron), mud logging reports, and regional stratigraphic correlations with Gulf of Suez and Red Sea rift models.

- Zeit Formation (Upper to Middle Miocene): Dominated by coarse-grained sandstones, conglomerates, and interbedded evaporites, reflecting a high-energy syn-rift depositional environment. Anhydrite indicates regional sealing capacity.
- South Gharib Formation (Upper to Middle Miocene): Thick halite sequences with minor shale and dolomite interbeds deposited under restricted evaporitic conditions.
- Belayim Formation (Middle Miocene): Sandstone–shale–carbonate alternations deposited in shallow marine settings with reservoir-quality sandstone units.
- Kareem Formation (Middle to Lower Miocene): Fluvial–deltaic to shallow marine sandstones interbedded with shales and carbonates.
- Rudeis Formation (Lower Miocene): Deep marine marly shales with interbedded siltstones and sandstones with potential source intervals.

### Source rock evaluation and hydrocarbons detection

#### Source rock potential: TOC and Rock-Eval pyrolysis

**Fig. 8:**
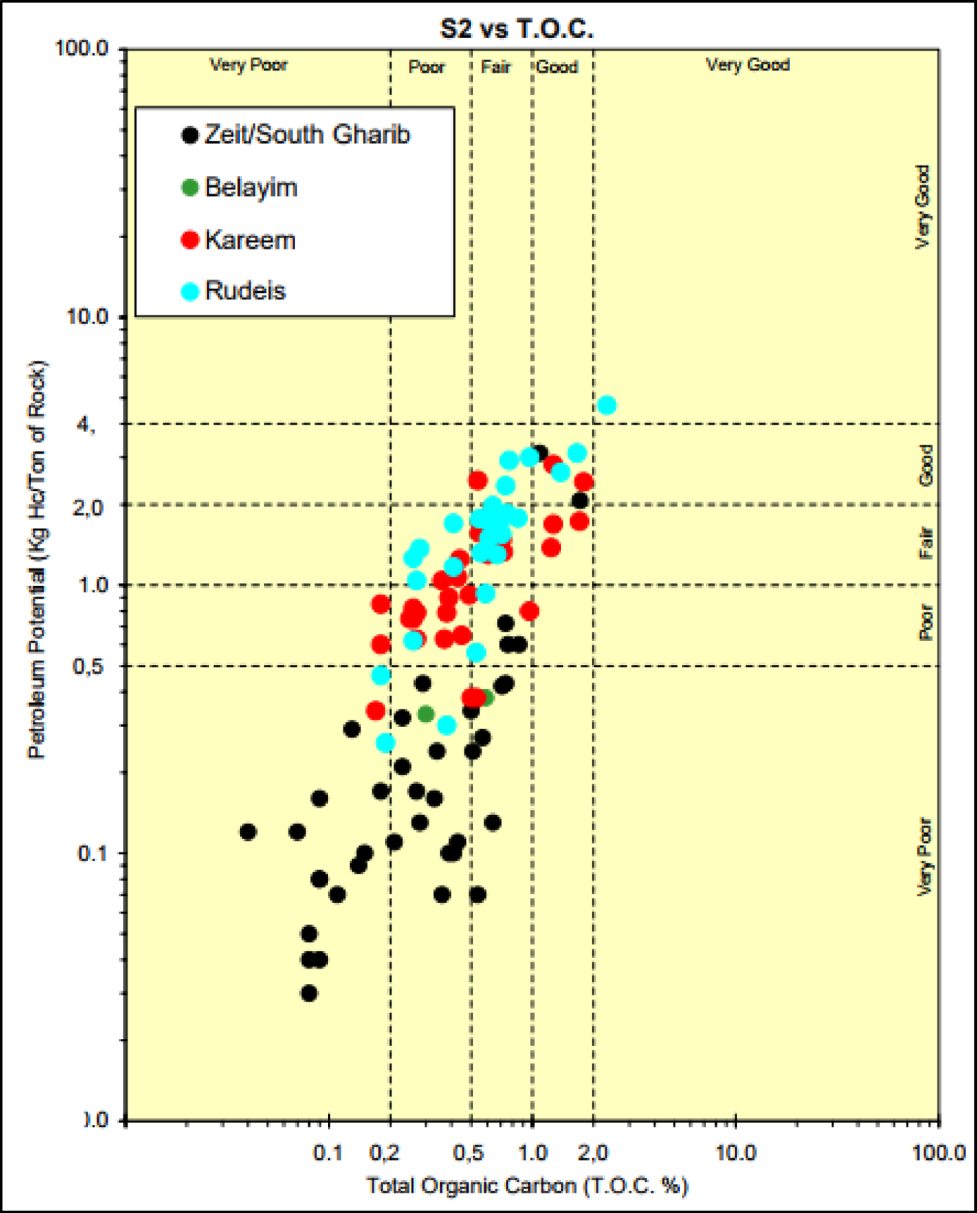
TOC of MIKAWA-1 WELL.

**Fig. 9:**
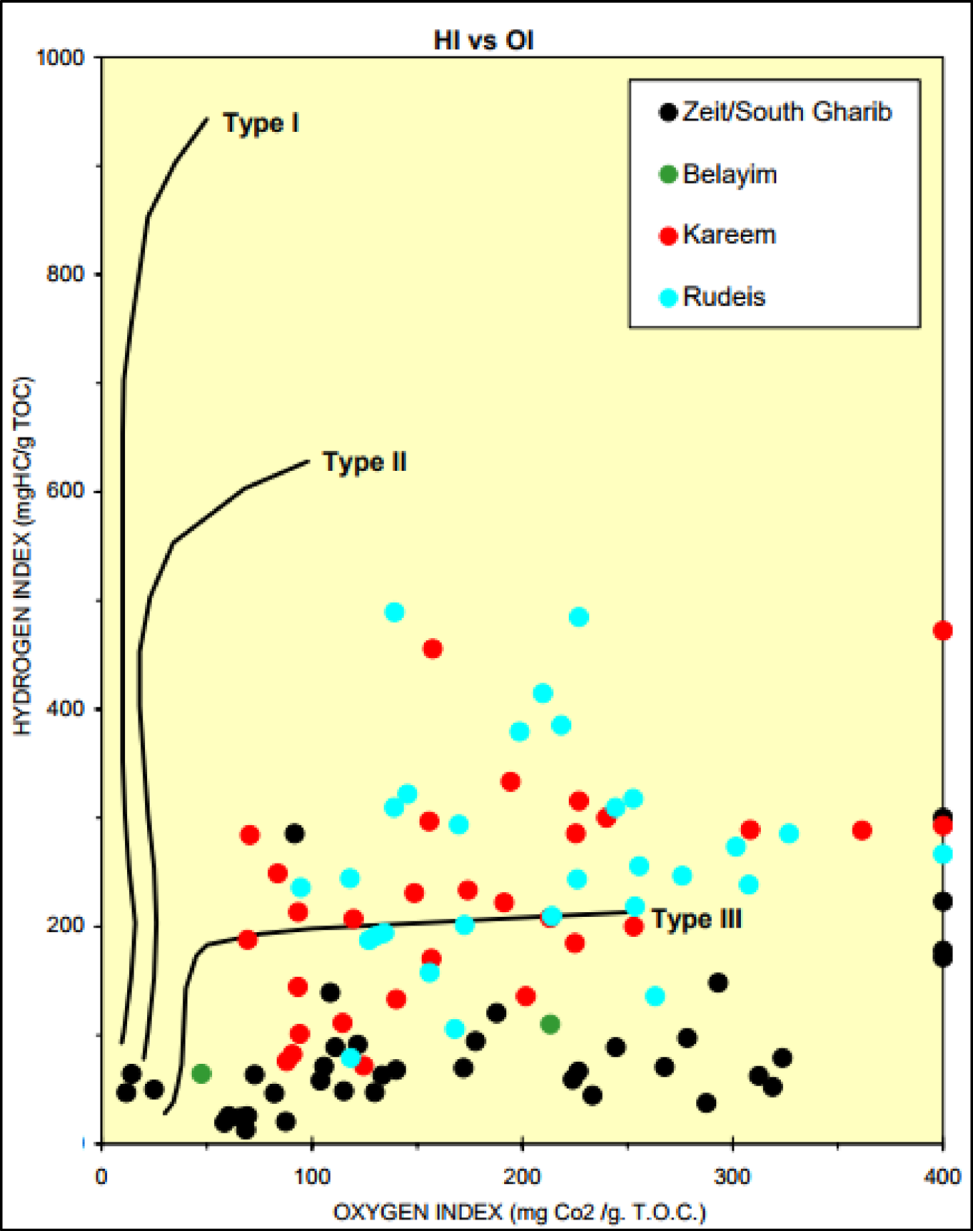
Rock Eval MIKAWA-1 WELL.

Rock–Eval pyrolysis was conducted on cuttings samples to evaluate source rock quality and hydrocarbon generation potential. Because the analyzed samples were primarily derived from drill cuttings, all datasets were screened for drilling additive contamination using S1–S2 peak morphology, Tmax consistency, biomarker distributions, solvent extraction behavior, and cross-validation with geochemical logs. (Figures 8 and 9).

The Zeit/South Gharib interval is characterized by generally low TOC values (< 0.5%, average 0.39%), negligible S2 yields (< 0.5 kg HC/ton rock), and HI values typically below 100 mg HC/g TOC, indicating poor source rock potential. A single interval at 2100 m shows relatively elevated TOC (1.09%) and S2 (3.11 kg HC/ton rock), although interpretation remains uncertain due to contamination effects.

Several samples, particularly at 2550 m, 2902 m, and 3001 m, display broadened S2 peaks and suppressed Tmax values (< 400 °C), consistent with drilling additive contamination rather than indigenous kerogen cracking. Consequently, these samples were excluded from direct source rock interpretation.

After filtering contaminated samples, the remaining dataset consistently indicates poor residual source rock quality. TOC values from the Belayim, Kareem, and Rudeis formations average 0.45%, 0.60%, and 0.67%, respectively. The integrated interpretation suggests limited remaining hydrocarbon generative capacity, indicating that source charge represents one of the principal exploration risks in the Northern Red Sea petroleum system.

#### Kerogen composition and maturity assessment

Kerogen typing and maturity evaluation were performed using optical microscopy and vitrinite reflectance (Ro) measurements. Nine samples between 3151 and 4171 m were analyzed.

The Zeit/South Gharib interval contains only trace organic matter, preventing reliable maturity determination. In contrast, the Kareem and Rudeis formations are dominated by Type III woody kerogen (80–90%) with minor herbaceous material, indicating predominantly gas-prone terrestrial organic matter deposited under deltaic to marginal marine conditions.

Thermal maturity interpretation reveals a significant contrast between Rock–Eval Tmax and vitrinite reflectance data. Although several Tmax values suggest immature to early mature conditions, measured Ro values range between 2.04% and 3.49%, indicating overmature conditions within the Lower Miocene interval. This discrepancy is interpreted as the result of drilling additive contamination and suppressed pyrolysis peaks rather than contradictory thermal evolution.

Accordingly, Ro measurements and biomarker maturity indicators were considered more reliable than Tmax for maturity assessment. The overmature nature of the Lower Miocene succession strongly limits present-day hydrocarbon generation potential and supports the interpretation of charge deficiency as a major exploration risk.

#### Hydrocarbon shows detected with rock-eval pyrolysis

Hydrocarbon indications were evaluated using S1 and Production Index (PI) data integrated with geochemical quality-control screening. Most samples exhibit low S1 and PI values, suggesting limited free hydrocarbons. Localized S1 enrichments below 3600 m coincide with abnormal S1/S2 ratios and contamination indicators and are therefore not interpreted as reliable hydrocarbon shows.

**Fig. 10:**
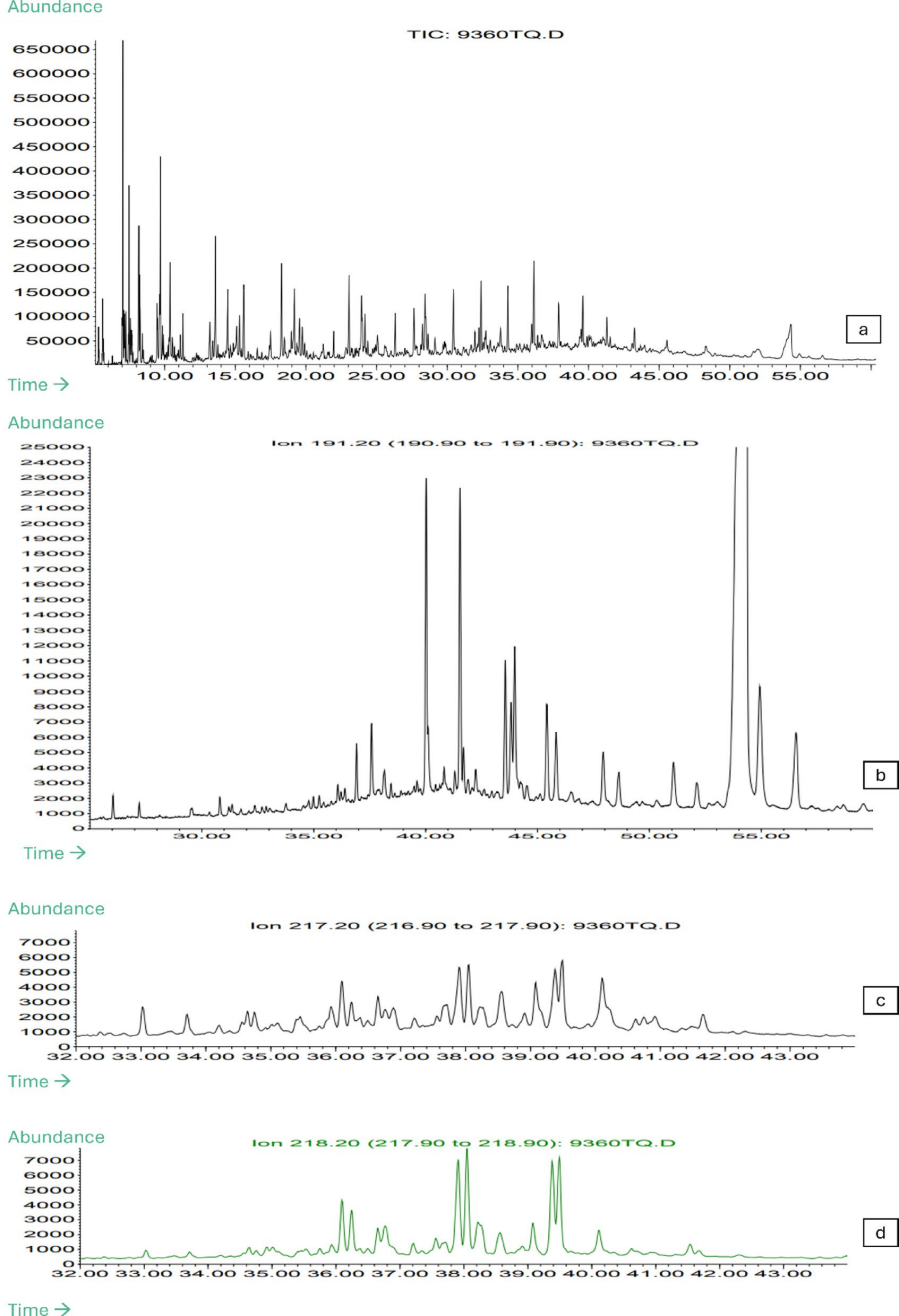
GC–MS chromatograms of sample 9360TQ.D showing molecular composition and biomarker distributions. (**a**) Total Ion Chromatogram (TIC) displaying overall compound abundance. (**b**) m/z 191 chromatogram highlighting terpane distributions. (**c**) m/z 217 chromatogram illustrating sterane distributions. (**d**) m/z 218 chromatogram showing sterane isomer variations within the 32–43 min retention interval.

To further evaluate hydrocarbon occurrence, solvent extraction, GC–MS, and isotopic analyses were conducted on 11 selected samples showing anomalous Rock–Eval responses (Fig. 10). These analyses confirm the presence of migrated hydrocarbons within several intervals, indicating that hydrocarbon migration occurred locally despite poor overall source rock quality.

#### Oil show characteristics

GC–MS biomarker analysis indicates the presence of migrated hydrocarbons within several intervals. The extract at 1960 m is characterized by elevated C30 hopane abundance, moderate gammacerane, C27–C28 sterane dominance, and low oleanane content, suggesting a mixed carbonate–shale source deposited under relatively reducing conditions.

The 2100 m extract displays similar biomarker characteristics but lower thermal alteration, suggesting a more local or indigenous hydrocarbon contribution. In contrast, the extracts at 3040 m and 3100 m show extended n-alkane distributions, reduced gammacerane abundance, low Pr/Ph ratios, and higher thermal maturity indicators, supporting migration from a mature petroleum system.

Although these biomarker signatures suggest possible linkage to Miocene–Upper Cretaceous source intervals, oil–source correlation remains preliminary because comprehensive regional quantitative biomarker datasets are unavailable. Nevertheless, the presence of migrated hydrocarbons demonstrates that hydrocarbon generation and migration occurred regionally, even if preservation efficiency was limited by trap and seal integrity.

### Reservoir analysis

Reservoir evaluation was based on integrated interpretation of density–neutron logs, resistivity logs, and mud logging data. Potential reservoir intervals were identified mainly within the Belayim, Kareem, and Rudeis formations.

Average porosity values are approximately 6%, 7.5%, and 9%, respectively, with net sand thicknesses of 12.1 m, 68.7 m, and 28.5 m. Although these values indicate only marginal reservoir quality by conventional standards, they remain regionally significant considering the deep burial and compaction effects affecting Northern Red Sea reservoirs.

Reservoir quality is highly heterogeneous and controlled primarily by facies distribution and syn-rift accommodation space. QUSEIR_B_1X lacks effective reservoir intervals due to dominance of evaporitic and shaly facies with consistently low effective porosity and high shale volume. This confirms reservoir absence as the principal failure mechanism in this well.

### Cap/Seal analysis

The Zeit and South Gharib evaporite sequences form the principal regional sealing system in the study area. Their lithological composition and lateral continuity provide strong sealing capacity; however, seismic interpretation indicates that seal integrity is locally compromised by fault systems.

The coexistence of thick evaporites and active faulting creates a dual control on hydrocarbon preservation. While evaporites provide effective vertical sealing in structurally stable areas, fault-related breach zones likely enhance vertical leakage and explain localized hydrocarbon shows within evaporitic intervals. Therefore, seal effectiveness is controlled primarily by structural integrity rather than lithology alone.

### Trap/Structure analysis

**Fig. 11:**
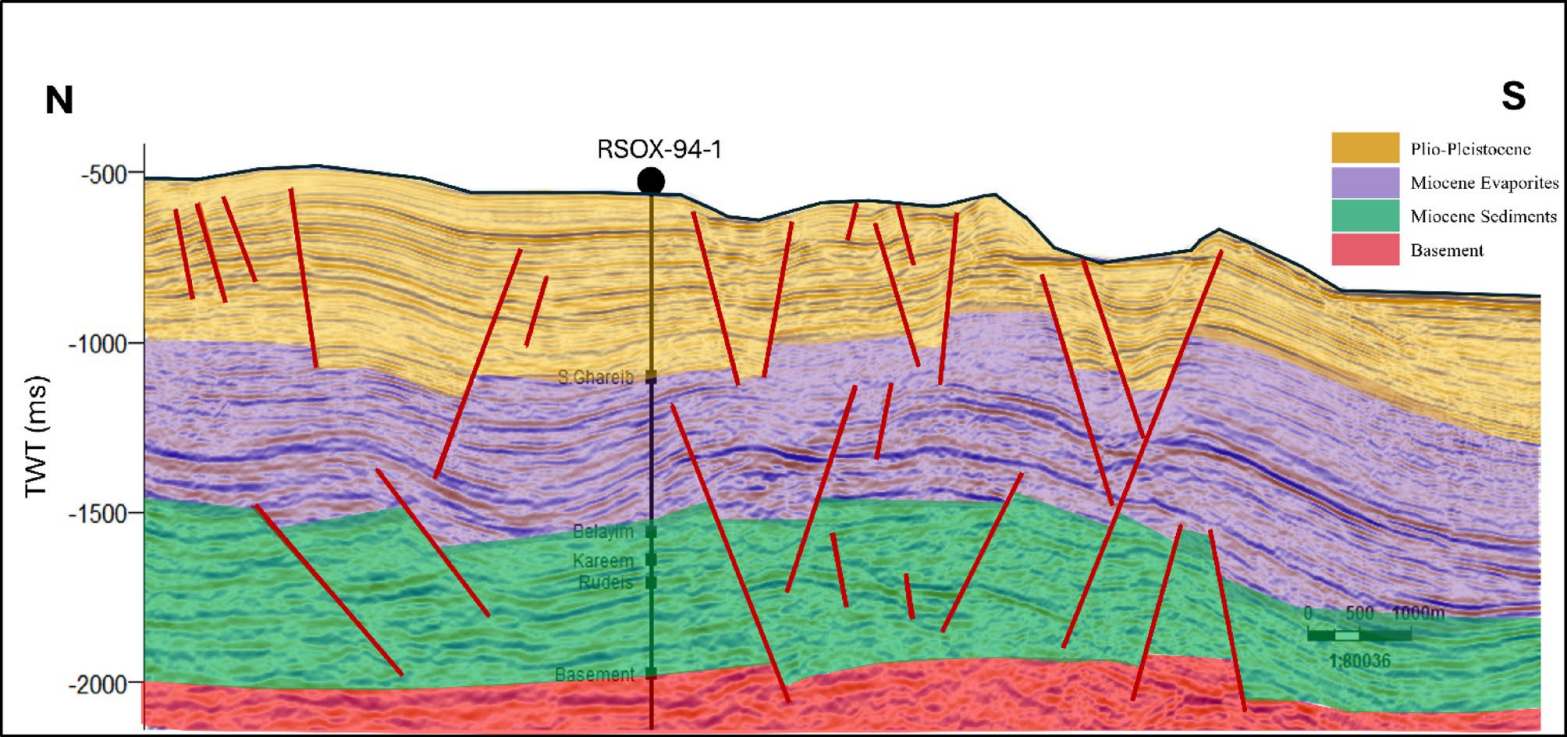
Interpreted seismic section showing numerous faults affecting the subsurface layers.

**Fig. 12:**
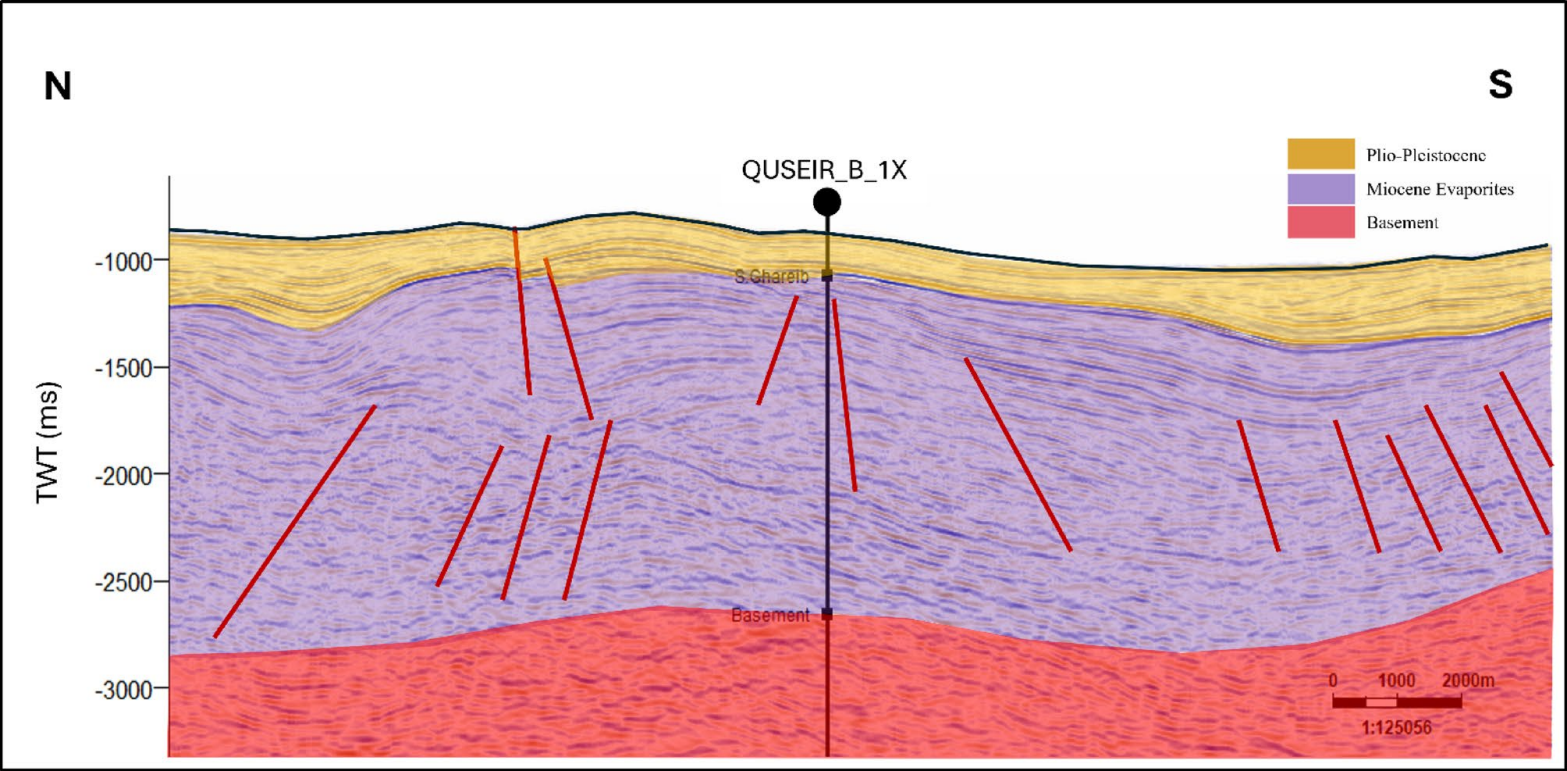
Interpreted seismic section showing well no QUSIER_B_1X encountered a complete section of evaporites and basements with the absence of Miocene reservoirs.

Seismic interpretation reveals that the structural framework of the Northern Red Sea is dominated by NW–SE trending tilted fault blocks associated with Red Sea rifting (Figs. 11 and 12). These structures represent the principal trapping geometries within the study area.

Structural interpretation was performed using 2D post-stack migrated seismic profiles calibrated with well data and regional stratigraphic markers. Fault mapping identified relay structures, compartmentalization zones, and fault systems capable of compromising seal integrity.

However, seismic interpretation beneath Miocene evaporites remains highly uncertain because salt-induced attenuation significantly reduces imaging quality in sub-salt intervals. Consequently, structural interpretation in deeper intervals relies heavily on well calibration and regional tectonic analogues.

The results indicate that trap presence alone is insufficient for successful hydrocarbon accumulation. Instead, preservation efficiency depends strongly on fault seal behavior and migration timing relative to structural evolution. This explains why structurally favorable traps such as RSOX-94-1 still failed due to seal breach.

### Failure reasons for each well

The integrated Dry Hole Analysis (DHA) demonstrates that each well failed due to a distinct petroleum system limitation (Table 1).

**Table 1:** Principal failure mechanisms identified from Dry Hole Analysis (DHA).

Well Name	Dominant Petroleum System Failure	Geological Interpretation	Exploration Implication
RSOX-94-1	Seal failure	Fault-related breach of evaporite seal	Future drilling should target structurally isolated fault blocks with improved seal integrity
MIKAWA-1	Charge failure	Overmature gas-prone source intervals with limited remaining generative capacity	Exploration should focus on areas with optimized thermal maturity and preserved charge systems
QUSEIR_B_1X	Reservoir failure	Absence of effective reservoir facies due to evaporitic/shaly deposition	Reservoir prediction should incorporate detailed facies and depositional modeling

### Integrated dry hole analysis

The integrated DHA demonstrates that exploration failure in the Northern Red Sea is multi-factorial and controlled by interactions between charge, reservoir, seal, and trap elements rather than by a single geological factor.

The results indicate that:

- Source charge deficiency represents the dominant regional-scale risk,
- Seal failure is the principal structural risk,
- and reservoir absence is a localized facies-controlled risk.

Although the structural framework provides numerous tilted fault block traps analogous to productive Gulf of Suez systems, effective hydrocarbon preservation remains limited due to fault-related leakage and overmature source intervals.

From an exploration perspective, the results emphasize that future drilling success will depend on:

- Identifying areas with optimal maturity windows,
- Targeting fault blocks with preserved seal integrity,
- Improving sub-salt seismic imaging through high-resolution 3D seismic acquisition,
- and integrating basin modeling with quantitative biomarker analysis to constrain migration timing and charge efficiency.

### Analogue prospects in the Red Sea: implications from Saudi Arabia and Yemen

Hydrocarbon production from Red Sea basins in Saudi Arabia and Yemen confirms the presence of a working regional petroleum system. These analogues demonstrate that faulted structural traps, Miocene evaporite seals, and syn-rift reservoirs can coexist within productive hydrocarbon systems.

However, the Northern Egyptian Red Sea differs in terms of maturity evolution, structural compartmentalization, and reservoir distribution. Therefore, regional analogues are used here primarily to constrain exploration concepts rather than establish direct source–trap correlations.

The integrated results suggest that future exploration should prioritize structurally preserved sub-salt traps with moderate maturity levels and improved reservoir development. Additional 3D seismic acquisition, basin modeling, and quantitative oil–source correlation studies will be critical for reducing exploration uncertainty in the Northern Red Sea.

## Conclusion

This study applied an integrated Dry Hole Analysis (DHA) workflow to evaluate the causes of exploration failure in the Northern Egyptian Red Sea using 2D seismic interpretation, petrophysical evaluation, Rock–Eval pyrolysis, biomarker analysis, and petroleum system integration from the RSOX-94-1, MIKAWA-1, and QUSEIR_B_1X wells. The results demonstrate that dry hole outcomes are controlled by the combined interaction of source, reservoir, seal, and structural elements rather than by a single geological factor.

Lithological and seismic interpretations indicate that the study area is dominated by syn-rift Miocene successions consisting of evaporites, sandstones, shales, and carbonates deposited within structurally controlled basins. The Zeit and South Gharib formations provide regionally extensive evaporite seals, whereas the Belayim, Kareem, and Rudeis formations constitute the principal reservoir and potential source intervals.

Geochemical evaluation reveals generally poor residual source rock quality throughout the analyzed intervals. Most TOC values are below 0.7%, while filtered S2 values are commonly less than 0.5 kg HC/ton rock, indicating limited remaining hydrocarbon generative potential. Because the analyzed samples were derived primarily from drill cuttings, a rigorous geochemical quality-control workflow was implemented to identify contamination associated with drilling additives. Integration of Rock–Eval screening, optical kerogen analysis, vitrinite reflectance, solvent extraction, and biomarker interpretation demonstrated that several anomalous pyrolysis responses were contamination-related rather than indicative of effective source rock potential.

Thermal maturity analysis indicates that the Lower Miocene succession is predominantly overmature, with vitrinite reflectance values ranging from 2.04% to 3.49%. Although localized hydrocarbon shows and migrated oil traces were identified through GC–MS analysis, the petroleum system appears to have experienced limited preservation efficiency because of overmature source intervals and reduced charge effectiveness.

Reservoir evaluation shows that the Belayim, Kareem, and Rudeis formations possess marginal to moderate reservoir quality, with average porosity values ranging from 6% to 9%. Reservoir distribution is strongly controlled by facies variability, burial compaction, and syn-rift structural development. QUSEIR_B_1X failed primarily because of the absence of effective reservoir facies.

Structural interpretation demonstrates that the Northern Red Sea contains numerous NW–SE-trending tilted fault blocks analogous to productive Gulf of Suez systems. Nevertheless, exploration success remains strongly dependent on trap preservation and charge effectiveness. In RSOX-94-1, hydrocarbon leakage associated with fault-related seal failure represents the principal failure mechanism, whereas MIKAWA-1 failed mainly because of insufficient hydrocarbon charge linked to overmature, gas-prone source rocks.

The integrated DHA results indicate that the dominant regional exploration risks are:

1. Insufficient hydrocarbon charge resulting from overmature source intervals.
2. Fault-related seal breach and hydrocarbon leakage.
3. Localized absence of effective reservoir facies.

Despite these challenges, the presence of migrated hydrocarbons, structurally favorable fault blocks, and productive Red Sea analogues in Saudi Arabia and Yemen confirms the existence of an active regional petroleum system.

Future exploration efforts should focus on:

- Identifying areas within optimal thermal maturity windows.
- Targeting structurally preserved fault blocks with intact evaporite seals.
- Improving prospect definition through targeted high-resolution 3D seismic surveys.
- Integrating basin modeling with quantitative oil–source correlation studies to better constrain hydrocarbon generation, migration pathways, and charge timing.

Overall, this study provides a petroleum system-based framework for understanding dry hole outcomes in the Northern Egyptian Red Sea and contributes to reducing geological uncertainty in future frontier exploration programs within this underexplored rift basin.

## Data Availability

The data that support the findings of this study are available from (The Egyptian General Petroleum Cooperation) but restrictions apply to the availability of these data, which were used under license for the current study, and so are not publicly available. This data is available from the corresponding author upon reasonable request and with permission of (The Egyptian General Petroleum Cooperation).
